# Predicting short- to medium-term care home admission risk in older adults: a systematic review of externally validated models

**DOI:** 10.1093/ageing/afae088

**Published:** 2024-05-10

**Authors:** Leonard Ho, Carys Pugh, Sohan Seth, Stella Arakelyan, Nazir I Lone, Marcus J Lyall, Atul Anand, Jacques D Fleuriot, Paola Galdi, Bruce Guthrie

**Affiliations:** Advanced Care Research Centre, Usher Institute, University of Edinburgh, Edinburgh, UK; Advanced Care Research Centre, Usher Institute, University of Edinburgh, Edinburgh, UK; Advanced Care Research Centre, Usher Institute, University of Edinburgh, Edinburgh, UK; Advanced Care Research Centre, Usher Institute, University of Edinburgh, Edinburgh, UK; Royal Infirmary of Edinburgh, NHS Lothian, Edinburgh, UK; Centre for Population Health Sciences, Usher Institute, University of Edinburgh, Edinburgh, UK; Royal Infirmary of Edinburgh, NHS Lothian, Edinburgh, UK; Centre for Cardiovascular Science, University of Edinburgh, Edinburgh, UK; Advanced Care Research Centre, Usher Institute, University of Edinburgh, Edinburgh, UK; School of Informatics, University of Edinburgh, Edinburgh, UK; School of Informatics, University of Edinburgh, Edinburgh, UK; Advanced Care Research Centre, Usher Institute, University of Edinburgh, Edinburgh, UK

**Keywords:** aged, long-term care, risk, validation study, systematic review, older people

## Abstract

**Introduction:**

Predicting risk of care home admission could identify older adults for early intervention to support independent living but require external validation in a different dataset before clinical use. We systematically reviewed external validations of care home admission risk prediction models in older adults.

**Methods:**

We searched Medline, Embase and Cochrane Library until 14 August 2023 for external validations of prediction models for care home admission risk in adults aged ≥65 years with up to 3 years of follow-up. We extracted and narratively synthesised data on study design, model characteristics, and model discrimination and calibration (accuracy of predictions). We assessed risk of bias and applicability using Prediction model Risk Of Bias Assessment Tool.

**Results:**

Five studies reporting validations of nine unique models were included. Model applicability was fair but risk of bias was mostly high due to not reporting model calibration. Morbidities were used as predictors in four models, most commonly neurological or psychiatric diseases. Physical function was also included in four models. For 1-year prediction, three of the six models had acceptable discrimination (area under the receiver operating characteristic curve (AUC)/*c s*tatistic 0.70–0.79) and the remaining three had poor discrimination (AUC < 0.70). No model accounted for competing mortality risk. The only study examining model calibration (but ignoring competing mortality) concluded that it was excellent.

**Conclusions:**

The reporting of models was incomplete. Model discrimination was at best acceptable, and calibration was rarely examined (and ignored competing mortality risk when examined). There is a need to derive better models that account for competing mortality risk and report calibration as well as discrimination.

## Key Points

- It is difficult for individual clinicians to accurately estimate care home admission risk.
- We critically appraised the existing five external validation studies of nine care home admission risk prediction models.
- Morbidities were used as predictors in four of the nine models, most commonly neurological or psychiatric diseases.
- For 1-year prediction, three of the six models had acceptable discrimination and the remaining three had poor discrimination.
- The only study examining model calibration, despite ignoring competing mortality, concluded that it was excellent.

## Introduction

Rapid population ageing is increasing the demand for health and social care. [1] Population ageing drives increasing prevalence of multimorbidity and geriatric syndromes (such as frailty, falls, continence problems and dementia) and increasing demand for care at home and residential care (which is variably named depending on country, like ‘care homes’, ‘nursing homes’ or ‘long-term care facilities’; this paper uses ‘care homes’). [1] Most health and social care systems aim to maintain independence at home for as long as possible because this aligns with most (but not all) individuals’ preferences, and it is usually less costly than residential care. Given constrained resources, targeting interventions at people at the highest risk of care home admission has the potential to maximise independence and ensure that any transition to residential care is agreed upon and planned rather than driven by an emergency. However, it is difficult for individual clinicians to accurately estimate care home admission risk, especially for patients with high risk of competing mortality (i.e. dying before care home admission). [2] This has driven interest in using formal prediction models to identify older adults at the highest risk of care home admission.

Several reviews have examined individual characteristics associated with care home admission since the 1980s. Wingard *et al.* synthesised cross-sectional and prospective studies of predictors of care home utilisation published before 1985. [3] They identified that age, sex, availability of caregivers and functional status were the predictors most commonly found to be significantly associated with care home admission. [3] A similar review by Luppa *et al.* two decades later found strong evidence for associations between care home admission and age, self-rated health status, functional and cognitive impairment, dementia, prior care home admission and number of prescriptions. [4] A smaller number of studies have attempted to derive formal models to predict care home admission using a variety of predictors, including the presence of various morbidities, physical function and professional judgement. Before any prediction model can be recommended for use in clinical practice, it requires external validation in a different dataset, target population or setting than the one used for model derivation. [5, 6]

Previous systematic reviews have synthesised and appraised models developed for predicting adverse outcomes in older adults, such as care home mortality, [7] emergency hospital admission, [8] hospital delirium, [9] and mortality (among community-dwelling participants). [10] They found that many of these prediction models did not have acceptable predictive performance, and their validations were often at risk of bias. The aim of this systematic review was therefore to evaluate external validation studies of prediction models for short- to medium-term care home admission risk (<3 years) in older adults aged ≥65 years.

## Methods

We conducted this review based on TRIPOD-SRMA (Transparent Reporting of multivariable prediction models for Individual Prognosis Or Diagnosis tailored for Systematic Reviews and Meta-Analyses) checklist [5] and PRISMA (Preferred Reporting Items for Systematic Reviews and Meta-Analyses) guidelines (Supplementary Table S1). [11] The review protocol was registered in PROSPERO (CRD42023410747).

### Eligibility criteria

Studies were eligible if they were prospective or retrospective cohort studies examining the external validation of models predicting care home admission risk over a time-horizon of 3 years or less, with the full text written in English. ‘Care home admission’ refers to the admission of the participant to a long-term care facility (i.e. institutions providing residential personal and/or nursing care) temporarily or permanently. Studies were eligible if they involved community-dwelling adults with average (mean or median) age ≥ 65 years, and were validated in people living in the community or at the point of hospital admission or emergency department (ED) attendance. Model predictors could be derived from electronic health record data, survey or trial data, data from questionnaires, other self-report assessment data and/or data from structured clinical assessment (e.g. comprehensive geriatric assessment). We included studies of well-established measures like Charlson Comorbidity Index, [12] either used as the only predictor or where the authors examined their performance with the addition of covariates (e.g. age and sex) not included in the core morbidity measure.

We excluded studies focusing only on specific populations (e.g. post-stroke, people with dementia). We also excluded conference abstracts, scoping, systematic and umbrella reviews, and clinical guidelines.

### Search strategy and selection criteria

We searched Medline, Embase and Cochrane Library from inception to 14 August 2023. Search strategies are defined in Supplementary Box S1, with additional hand-searching of reference lists of included studies and excluded conference abstracts. We imported all records into Covidence (https://www.covidence.org/) (Veritas Health Innovation, Melbourne, Australia) with title and abstract screening done by two reviewers (L.H. and B.G.), and full-text screening completed by one reviewer (L.H.) and then validated by another reviewer (B.G.).

### Data extraction and risk of bias and applicability assessment

We used CHARMS (CHecklist for critical Appraisal and data extraction for systematic Reviews of prediction Modelling Studies) to extract the characteristics of included studies and their prediction models. [13] The study characteristics included first author, publication year, study location, funding source, study design, use of collected data, source of data, outcome definition, measurement of outcome, participant selection criteria, number of participants, age, sex, and race or ethnicity of participants and number of admissions. The model characteristics included statistical modelling method used in model development (retrieved from previous papers reporting model development), number of predictors, types of predictors, time of prediction, prediction time-horizon, reported performance measures and whether model performance measures accounted for competing mortality. Performance measures extracted included measures of discrimination (e.g. area under the receiver operating characteristic curve (AUC), Harrell’s *c* statistic, and metrics such as sensitivity and specificity at selected cut-points, calibration (e.g. calibration plot), and measures of overall performance, reclassification and clinical usefulness (e.g. pseudo-*R*^2^, net reclassification index and decision curve). [14] Discrimination measures how well the model distinguishes between people who are admitted to care homes and people who are not. Calibration reflects the agreement between observed and expected events (i.e. are predictions accurate), and is a critical performance feature for clinical use. A prediction model may have good discrimination in terms of predicted risk being higher in those admitted to care homes versus those not but produce predictions which are poorly calibrated (inaccurate).

We conducted risk of bias and applicability assessment for the validation studies of prediction models using PROBAST (Prediction model Risk Of Bias Assessment Tool). [15] The above procedures were performed by one reviewer (L.H.) and then independently validated by another (B.G.). Disagreements were resolved by discussion between the two reviewers.

### Data synthesis

No models were externally validated more than once, with high between-study heterogeneity, and meta-analysis to estimate pooled discrimination was therefore not appropriate. Instead, we narratively synthesised findings using descriptive statistics and tables. We adopted commonly used cut-off points for discrimination to aid interpretation, by considering a prediction model with AUC or *c s*tatistic between 0.50 to 0.69 as having poor discrimination, 0.70 to 0.79 acceptable discrimination, 0.80 to 0.89 excellent discrimination and ≥ 0.90 outstanding discrimination (for these measures, a value of 0.50 means the model performs no better than chance, and 1.00 means that discrimination is perfect). [16, 17] No generally agreed cut-off points have yet been available for sensitivity and specificity, likelihood ratios (LRs) or predictive values. The results were reported according to the prediction time-horizon which varied between 1 month and 1 year, with one study reporting model performance over two time-horizons. Where authors calculated discrimination using two or more sources of data (e.g. inpatient data only versus inpatient and outpatient data), we used the best results to summarise model performance. As calibration is harder to formally assess because it involves more judgement, [18] we extracted the authors’ summary interpretation of model calibration.

## Results

### Study selection

**Figure 1:**
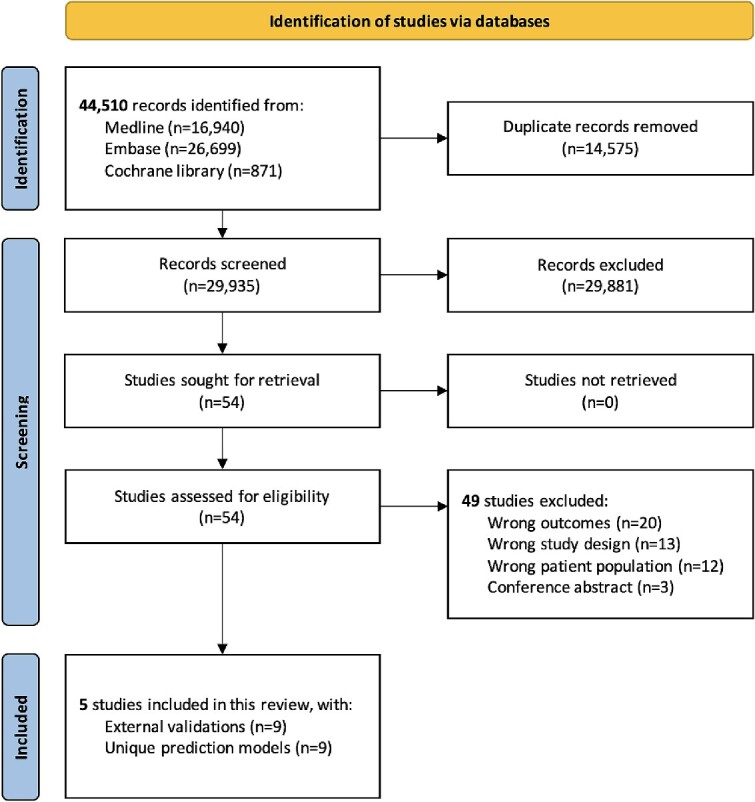
Flow of literature search and selection

The literature search yielded 44,510 records. After deduplication, we performed title and abstract screening on 29,935 records, of which 54 full-text records were screened (Figure 1). Five studies were eligible, reporting external validation of nine unique prediction models.

### Study characteristics

Table 1 details the characteristics of the included studies. The five included studies were published between 2005 and 2023, with two published in Canada [19, 20] and one each in the USA, [21] Ireland, [22] and Switzerland. [23] Two studies were funded by governments and/or other public bodies, [19, 23] one was supported by a private research institute, [21] and two did not report on their funding sources. [20, 22] Three of the included studies were retrospective cohort studies. [20–22] Three studies externally validated existing models, [19, 20, 23] and two developed and external validated new models. [21, 22] Two studies used data obtained from clinical assessments and staff-administered questionnaires, [19, 23] two used previous survey or trial data, [20, 22] and one used electronic health record data. [21] Prediction models were validated in a total of 5,343,487 participants, but most validation studies were relatively small (median 444; interquartile range 2,671,208.5), with average age (mean or median) ranging from 74.0 to 85.3 years (if reported). Only one study reported participants’ race or ethnicity. [21] One study involved participants aged <65 years. [21].

**Table 1:** Characteristics of the included studies

Author (year)	Study location (funding source(s))	Study design (use of collected data)	Data source(s)	Outcome definition (measurement of outcome)	Participants eligibility criteria	Number of participants	Participant age, sex and race/ethnicity	Number of admissions (%)
Fan (2006)	Canada (Funded by the St Peter’s Hospital Regional Geriatric Programme and The Canadian Association of Emergency Physicians)	Prospective cohort (External validation of an existing model)	Clinical assessments Questionnaires administered by staff	Any permanent change of residence into a long-term care facility (Obtained from hospital administrative and community-wide databases and charts)	Inclusion: aged ≥65 years; and consecutively accessing the academic ED without inpatient admission Exclusion: residents of a long-term care facility; previously enrolled in this study or if cognitively impaired and simultaneously had no available proxy to answer the screening questions	120 (1 died and 2 lost-to-follow-up within 1 month; 4 died and 3 lost-to-follow-up within 4 months)	65 to 69 years 17.5%, 70 to 74 years 25.0%, 75 to 79 years 29.2%, 80 to 84 years 19.1%, ≥85 years 9.2% (Median/mean age not reported) Female: 55.9%	0 (0) at 1 month^a^ 3 (2.5) at 4 months
Greenwald (2022)	United States (Funded by the Health Data Analytics Institute)	Retrospective cohort (Development and external validation of a new model)	Electronic health records	Discharged to a location other than home (with or without organised home health care) (Obtained from the Medicare fee-for-service and dual-eligible (Medicaid and Medicare) files)	Inclusion: aged ≥18 years; and hospitalised Exclusion: aged <18 years; aged >99 years; had missing records or inconsistent data; had either discontinuous Part A or Part B Medicare coverage; or had Part C coverage in the year before admission	5,336,265 (149,415 died in hospital during follow-up)	Median age: 74.0 Female: 54% White 81.7%, Black 11.7%, Asian 1.4%, Other 5.0%	1,915,719 (35.9)
Mayo (2005)	Canada (Sources not reported)	Retrospective cohort (External validation of an existing model)	Previous survey or trial data)	Admitted to a long-term care facility (Obtained from the Régie de l’assurance maladie du Québec, the Québec Ministry of Health and Social Services, and the MedEcho database)	Inclusion: aged ≥65 years; and participated in a previous trial (MOXXI-I) Exclusion: not reported	6465 (133 died during follow-up)	Mean age: 75.1 Female: 63.0%	61 (0.9)
O’Caoimh (2023)	Ireland (Sources not reported)	Retrospective cohort (Development and external validation of a new model)	Previous survey or trial data	Admitted to a nursing home (providing low or high levels of dependency on activities of daily living) excluding those living in sheltered accommodation (i.e. assisted living or supportive housing programmes or in retirement communities) (Obtained from the hospital’s Patient Administration System and from the county’s local placement forum)	Inclusion: community-dwelling; aged ≥70 years; visited the ED; and with MTS score > 1 Exclusion: in an unstable medical condition according to the MTS (i.e. a score of one); nursing home residents; admitted directly to intensive care or the cardiac care unit; or in long-term residential care	193 (33 died during follow-up)	Median age: 79.0 Female: 55.0%	27 (13.5)
Zekry (2012)	Switzerland (Funded by the Swiss National Science Foundation and the Swiss Foundation for Ageing Research)	Prospective cohort (External validation of an existing model)	Clinical assessments Questionnaires administered by staff	Permanently admitted to a long-term care institution (Obtained through phone calls to the patient, family and/or general practitioner, or through access to the population registrar of the State of Geneva)	Inclusion: aged ≥75 years; and consecutively admitted to geriatric inpatient units Exclusion: not reported	444 (97 died during follow-up)	Mean age: 85.3 Female: 74.0%	124 (27.9)

ED: emergency department; MTS: Manchester Triage System score.

^a^0.5 was used (instead of 0) by the original authors for calculating the metrics of model discrimination.

Two studies predicted care home admission risk for ED attendees, one for general inpatients, one for geriatric service inpatients and one for people living in the community. All studies reported their definition of care home admission, but only two explicitly stated whether admissions were permanent and/or temporary (in both cases, only including permanent admissions). [19, 23] Care home admission was ascertained using routine administrative data, [20–22] routine data plus chart review, [19] or routine data plus phone calls to patients and professionals. [23] The percentage of participants admitted to a care home ranged from 0.9% of people participating in a clinical trial admitted to a ‘long-term care facility’ in Canada [20] to 35.9% of inpatients in the USA discharged to a location ‘other than home’. [21].

### Prediction model characteristics

Table 2 and Supplementary Table S2 detail the included prediction models and their validations. Seven of the nine models were developed using logistic regression, while the remaining two (‘Clinical Frailty Scale’ and ‘Geriatric Index of Comorbidity’) used survival analysis (Cox regression). [24, 25] The time-horizon over which prediction was examined ranged from 1 month to 1 year, but most evaluations were at 1 year. Six (66.7%) of the nine included models predicted risk in ED attendees, two (22.2%) during inpatient admission and one (11.1%) in people living in the community. The median number of predictors included in models was 5.5 (range 1 to 19; interquartile range 3 to 11), but one study did not report the number of predictors. [21]

**Table 2:** Characteristics of the included models

Author (year)	Prediction model	Statistical modelling method used in model development	Number of predictors	Time of prediction	Prediction time-horizon	Discrimination^a^	Calibration^b^	Accounted for competing mortality in calibration
Fan (2006)	Triage Risk Screening Tool for Elderly Patients	Logistic regression	5	During ED attendance	1 month 4 months	Not acceptable^c^ 1 month^d^ Positive LR 1.03 Negative LR 0.98 4 months Positive LR 1.81 Negative LR 0.98	Not reported	No
Greenwald (2022)	Risk Stratification Index 3.0	Logistic regression	Not reported	During inpatient admission	3 months	Acceptable AUC 0.79	Excellent	No
Mayo (2005)	Quan-Charlson Comorbidity Index with covariates	Logistic regression	19	Anytime in the community	1 year	Acceptable Harrell’s *c* statistic 0.72	Not reported	No
O’Caoimh (2023)	Clinical Frailty Scale	Cox regression	1	During ED attendance	1 year	Poor AUC 0.68	Not reported	No
O’Caoimh (2023)	Identification of Seniors At Risk	Logistic regression	6	During ED attendance	1 year	Poor AUC 0.64	Not reported	No
O’Caoimh (2023)	Programme of Research to Integrate Services for the Maintenance of Autonomy 7	Logistic regression	7	During ED attendance	1 year	Poor AUC 0.66	Not reported	No
O’Caoimh (2023)	Risk Instrument for Screening in the Community (Global score)	Logistic regression	3	During ED attendance	1 year	Acceptable AUC 0.70	Not reported	No
O’Caoimh (2023)	Risk Instrument for Screening in the Community (Overall score)	Logistic regression	3	During ED attendance	1 year	Acceptable AUC 0.73	Not reported	No
Zekry (2012)	Geriatric Index of Comorbidity	Cox regression	15	During inpatient geriatric service admission	1 year	Acceptable^e^ Specificity 99.7% PPV 50.0% NPV 72.2%	Not reported	No

AUC: area under the receiver operating characteristic curve; ED: emergency department; PPV: positive predictive value; NPV: negative predictive value; LR: likelihood ratio.

^a^Poor discrimination refers to AUC or Harrell’s *c* statistic between 0.50 to 0.69; acceptable discrimination refers to AUC or Harrell’s *c* statistic between 0.70 to 0.79; excellent discrimination refers to AUC or Harrell’s *c* statistic 0.80 to 0.89; outstanding discrimination refers to AUC or Harrell’s *c* statistic ≥0.90

^b^We extracted the authors’ summary interpretation of model calibration.

^c^Based on the original authors’ judgement with justification (model not clinically useful due to small LRs).

^d^The number of admissions (event) was 0, and therefore the original authors used 0.5 to calculate the metrics of model discrimination.

^e^Based on the original authors’ judgement with justification (model predicts accurately due to adequate metrics).

Table 3 details the predictors used by the nine unique models. The most included types of predictors were morbidities (four models; 44.4%), physical function (four models; 44.4%) and professional judgement (i.e. nurses’ concerns or perceived chance of admission) (three models; 33.3%). The morbidities included varied considerably. Neurological or psychiatric diseases, such as cerebrovascular diseases and cognitive impairment, contributed to four (44.4%) prediction models, with three models using cancer, cardiovascular, respiratory, renal, metabolic, gastrointestinal and liver diseases as distinct predictors. Other conditions, such as visual impairment [21, 22] and musculoskeletal conditions, [21, 23] were adopted by some prediction models. ‘Programme of Research to Integrate Services for the Maintenance of Autonomy 7’ (PRISMA7) model also included estimates of general health and social support as predictors. [22]

**Table 3:** Predictors used in the included prediction models

Type of predictor	Number of models including the predictor type	Prediction model (Author, year)								
		Triage Risk Screening Tool for Elderly Patients (Fan 2016)	Risk Stratification Index 3.0 (Greenwald, 2022)	Quan-Charlson Comorbidity Index with covariates (Mayo, 2005)	Clinical Frailty Scale (O’Caoimh, 2023)	Identification of Seniors at Risk (O’Caoimh, 2023)	Programme of Research to Integrate Services for the Maintenance of Autonomy 7 (O’Caoimh, 2023)	Risk Instrument for Screening in the Community (Global score) (O’Caoimh, 2023)	Risk Instrument for Screening in the Community (Overall score) (O’Caoimh, 2023)	Geriatric Index of Comorbidity (Zekry, 2012)
Morbidities	4		●	●		●				●
Neurological/psychiatric	4		●	●		●				●
Cancer	3		●	●						●
Cardiovascular	3		●	●						●
Respiratory	3		●	●						●
Renal	3		●	●						●
Metabolic	3		●	●						●
Gastrointestinal	3		●	●						●
Liver	3		●	●						●
Urogenital/sexually transmitted	2		●	●						
Rheumatological	2		●	●						
Haematological	2		●							●
Visual impairment	2		●			●				
Musculoskeletal conditions	2		●							●
All other conditions in ICD-10	1		●							
Physical function	4	●			●	●	●			
Professional judgement	3	●						●	●	
Age	2			●			●			
Sex/gender	2			●			●			
Care requirements	2					●	●			
Previous admissions/length of stay	2	●				●				
Medications	2	●				●				
General health and social support	1					●				

ICD-10: Tenth edition of the International Classification of Diseases.

### Prediction model performance

#### Model discrimination

Overall, seven (77.8%) of the nine validation studies reported model discrimination as AUC or *c* statistic, while the remainder used LRs or specificity with predictive values, to illustrate the performance measure (Table 2 and Supplementary Table S2). One validation reported model discrimination at 1 month and 4 months, one at 3 months and the remaining seven at 1 year.

Among non-admitted ED attendees, ‘Triage Risk Screening Tool for Elderly Patients’ had a positive LR of 1.03 and negative LR of 0.98 at 1 month, compared with positive LR of 1.81 and negative LR of 0.98 at 4 months. The authors concluded that performance was not acceptable for clinical use. [19] Among hospitalised patients, ‘Risk Stratification Index 3.0’ had acceptable model discrimination at 3 months (AUC 0.79). [21] For 1-year prediction, three models reported acceptable model discrimination (AUC or *c* statistic 0.70–0.79) among community-dwelling participants [20] and non-admitted ED attendees. [22] Three reported poor model discrimination (AUC or *c* statistic <0.70) for the same prediction time-horizon. [22] The validation of ‘Geriatric Index of Comorbidity’ reported specificity (99.7%) but not sensitivity, positive predictive value (PPV) (50.0%), negative predictive value (NPV) (72.2%), concluding that the prediction model accurately predicts care home admission among those admitted to geriatric inpatient units. [23]

#### Model calibration

Only one (11.1%) of the nine validation studies reported calibration in any way (Table 2 and Supplementary Table S2). The validation of ‘Risk Stratification Index 3.0’ presented a calibration plot for prediction at 3 months, and calculated observed–expected ratio (0.98) and estimates of calibration intercept (0.00) and slope (1.00). The authors concluded that the model was ‘close to ideal’ (i.e. excellent) for most of the study population, [21] although examination of calibration did not account for competing mortality risk which may make this conclusion optimistic. [2, 26]

#### Other model performance measures

Only one (11.1%) validation examined other model performance measures (Supplementary Table S2). The validation of ‘Geriatric Index of Comorbidity’ calculated the pseudo-*R*^2^ (0.06) of the prediction model at 1-year but did not explicitly interpret the result. [23]

### Risk of bias and applicability of the validations

Overall, eight (88.9%) of the nine validation studies were at high risk of bias, [19, 20, 22, 23] with only one at low risk (Table 4 and Supplementary Figure S1). [21] All had satisfactory performance in the participants, predictors and outcome domains of PROBAST. However, in the analysis domain, those with high risk of bias did not report both model discrimination and model calibration. Seven (77.8%) validation studies also did not have ≥100 care home admissions (events) by the end of follow-up. [19, 20, 22] Applicability to the target population of older people was generally good, but one (11.1%) had unclear concerns over applicability because it included some participants aged <65 years. [21]

**Table 4:** Results of risk of bias and applicability assessment

Prediction model	Author, year	Risk of bias				Applicability			Overall	
		1. Participants	2. Predictors	3. Outcome	4. Analysis	1. Participants	2. Predictors	3. Outcome	Risk of bias	Applicability
Triage Risk Screening Tool for Elderly Patients	Fan, 2016	+	+	+	-	+	+	+	-	+
Risk Stratification Index 3.0	Greenwald, 2022	+	+	+	+	?	+	+	+	?
Quan-Charlson Comorbidity Index with covariates	Mayo, 2005	+	+	+	-	+	+	+	-	+
Clinical Frailty Scale	O’Caoimh, 2023	+	+	+	-	+	+	+	-	+
Identification of Seniors at Risk	O’Caoimh, 2023	+	+	+	-	+	+	+	-	+
Programme of Research to Integrate Services for the Maintenance of Autonomy 7	O’Caoimh, 2023	+	+	+	-	+	+	+	-	+
Risk Instrument for Screening in the Community (Global score)	O’Caoimh, 2023	+	+	+	-	+	+	+	-	+
Risk Instrument for Screening in the Community (Overall score)	O’Caoimh, 2023	+	+	+	-	+	+	+	-	+
Geriatric Index of Comorbidity	Zekry, 2012	+	+	+	-	+	+	+	-	+

+ indicates ‘low risk of bias’ for Risk of Bias assessment or ‘low concern over applicability’ for Applicability assessment.

? indicates ‘unclear risk of bias’ for Risk of Bias assessment or ‘unclear concern over applicability’ for Applicability assessment.

- indicates ‘high risk of bias’ for Risk of Bias assessment or ‘high concern over applicability’ for Applicability assessment.

## Discussion

This systematic review examined five studies reporting the validation of nine unique prediction models for risk of care home admission in older adults for a variety of prediction time-horizons up to 1 year. The overall risk of bias in the validation studies was generally high, with only one of them reporting both model discrimination and calibration. The prediction models examined used a wide variety of predictors, with 44.4% using morbidities and physical function, respectively. The most common group of morbidities used as predictors was neurological or psychiatric diseases, closely followed by cancer and cardiovascular, respiratory, renal, metabolic, gastrointestinal and liver diseases. For 1-year risk prediction, three of the seven prediction models had acceptable (but never good or excellent) model discrimination (AUC or *c s*tatistic 0.70 to 0.79), three had poor discrimination (AUC or *c s*tatistic < 0.70) and one was reported to be able to accurately predict the outcome based on high specificity. Only one validation explicitly evaluated model calibration, concluding it was excellent, although the outcome measured was ‘discharge to a facility’ is much broader than most other studies, was very common (35.9%) and very likely includes many temporary admissions, and therefore may not generalise to non-US health care systems.

Similar to our findings, external validation studies of models for predicting older adults’ risks of mortality if resident in care homes, [7] emergency hospital admission, [8] and general mortality [10] tended not to report model calibration, resulting in high risk of bias ratings in PROBAST assessment. Most of their prediction models failed to achieve excellent model discrimination (AUC or *c s*tatistic ≥ 0.80). Our findings are also consistent with the review of studies on the prediction models for care home admission risk in people aged ≥50 years (although not restricted to external validations), where even in internal validation, discrimination was poor or at best acceptable. [27]

Strengths of this systematic review include the performance of a comprehensive literature search in major electronic databases and reporting according to CHARMS and PROBAST. There are also some limitations. First, since the included prediction models were only externally validated once and there is high between-study heterogeneity, we could not perform meta-analyses to estimate pooled model discrimination results. Therefore, generalisability to a wider range of clinical contexts is uncertain, and generalisability between countries particularly so (because of differences in organisation and funding of care, and differences in informal care expectations and capacity). Second, our understanding of calibration is inadequate because only one validation study reported it. This is a major gap in the literature given the very wide variation in care home admission rates reported which likely arises from differences in outcome measurement including whether an admission is permanent or temporary, from differences in the population studied (community, all ED attendees, ED attendees who are not admitted, inpatients or trial participants), and from differences in how residential and home care is organised and funded in different countries. Third, most validation studies did not report their study population in detail to ensure that their findings apply to diverse populations, especially in terms of race and ethnicity. Fourth, the outcome in Greenwald *et al.* does not distinguish discharge to long-term care facilities from discharge to other health or social care facilities, such as skilled nursing facilities, and likely includes many temporary or ‘step-down’ admissions. [21] Therefore, readers should be cautious when comparing the performance of the ‘Risk Stratification Index 3.0’ to other models. Finally, restricting the analyses to external validation studies means that some potentially relevant prediction tools were not considered (e.g. ‘Electronic Frailty Index’ by Clegg *et al.* where discrimination was acceptable in internal validation). [28] However, prediction tool performance is typically worse in external validation, [29] and external validation is always recommended before clinical use.

Although three models had acceptable discrimination (a measure of the general ability to distinguish those admitted from those not), the lack of published data on calibration (whether predicted risk is accurate) means that the main clinical implication is that none of the tools can be strongly recommended for routine use. We believe that at best, existing tools could be used as screening instruments to identify patients for clinical review, but clinicians or policy-makers interested in using one of these tools should carefully consider whether a tool has been derived in a population similar to their own and ideally check calibration of predictions in their own population before clinical use. [30]

In terms of research, there is a need for broader external validation of existing models, and for the derivation and validation of better prediction models for care home admission risk in older adults. Future research might compare prediction models using different combinations of routine clinical data and bespoke data (e.g. clinical assessment or self-report of function) to clarify if models using additional bespoke data provide any performance benefit that justifies their additional cost of data collection. Model evaluation should also account for competing mortality, since not accounting for it will typically lead to models over-predicting risk of care home admission. [2] For existing and new models, future research should also focus on high-quality external validations that robustly examine model calibration and discrimination, [5] as well as on those that evaluate model performance in important subgroups (e.g. by age group, gender, race and ethnicity, or presence of common morbidities) because good overall performance may conceal poor performance in critical subgroups. [26] External validation studies should also include head-to-head comparisons of different prediction models in the same population to support the selection of models for clinical use. [30] All of these validation studies should be reported according to TRIPOD (Transparent Reporting of a multivariable prediction model for Individual Prognosis Or Diagnosis). [31]

More broadly, care home admission is a complex outcome compared with other outcomes of interest in older people like mortality, and service organisation varies considerably between countries, meaning that it is possible that prediction tools will need to be country or context specific. Even within countries, ‘admission’ can be intended to be permanent from the outset or intended to be short-term, and there are important differences between those admitted from the community versus from hospitals (who will often die soon after care home admission). [3] Prediction tool developers need to be explicit about (and justify) their choice of the context of prediction (community, ED or inpatient) and their choice of the outcome (permanent versus temporary), as well as the data limitations on how care home admission is measured. [32, 33] Finally, it is important to recognise that it is uncertain whether it is possible to create prediction tools for care home admission which are very high performing, because moving to care home may be dependent on a much wider range of individual factors than can be easily measured, meaning that there may be performance ceilings for models derived from routine data in particular. [21]

## Conclusions

This systematic review synthesised five external validations of nine unique prediction models for short- to medium-term care home admission risk in older adults. The risk of bias in the validation studies was generally high, the performance of the models in terms of discrimination was never better than adequate and their calibration was poorly reported. There is a need to develop and robustly validate better prediction tools to help identify older adults at high risk of care home admission to inform the delivery of health and social care interventions to promote independent living.

## Supplementary Material

aa-23-1642-File002_afae088
