# Supplementary material for: Predicting short- to medium-term care home admission risk in older adults: a systematic review of externally validated models
Source: Age Ageing. 2024 May 10;53(5):afae088. doi: 10.1093/ageing/afae088 (PMC11084757; doi:10.1093/ageing/afae088)
Supplement: aa-23-1642-File002_afae088 [file aa-23-1642-file002_afae088.docx]

**Prediction models for predicting short- to medium-term care home admission risk in older adults: a systematic review of externally validated models**

**Supplementary file**

**Box S1.** Search strategies for databases

**Figure S1.** Summary of risk of bias and applicability assessment

**Table S1.** PRISMA (Preferred Reporting Items for Systematic Reviews and Meta-Analyses) checklist

**Table S2.** Further details on model characteristics

**Box S1.** Search strategies for databases

**Medline:**

1. chronic disease scor*.ti,ab.

2. mortality risk* ind*.ti,ab.

3. ((charlson* or elixhauser* or comorbid* or co-morbid*) adj2 (index or indices)).ti,ab.

4. cumulative illness rating scale*.ti,ab.

5. adjusted clinical group*.ti,ab.

6. (risk* adj2 (tool* or index or indices or score* or scale* or predict*)).ti,ab.

7. ((prognos* or predict*) adj2 (tool* or index or indices or score* or scale*)).ti,ab.

8. 1 or 2 or 3 or 4 or 5 or 6 or 7

9. valid*.ti,ab.

10. 8 and 9

11. (rxrisk* or rx risk*).ti,ab.

12. (medication* adj3 burden* ind*).ti,ab.

13. burden of illness scor*.ti,ab.

14. functional morbidity ind*.ti,ab.

15. multidimension* prognos* ind*.ti,ab.

16. silver code.ti,ab.

17. health intelligence system*.ti,ab.

18. combined predict* model*.ti,ab.

19. hospital admission risk profile*.ti,ab.

20. (predict* emergency admission* adj3 next year*).ti,ab.

21. predictive risk stratification model*.ti,ab.

22. (qadmission* or q-admission*).ti,ab.

23. (sparra or “scottish patients at risk of readmission and admission” or “scottish patients at risk of re-admission and admission”).ti,ab.

24. sussex predictor of key event*.ti,ab.

25. (“patients at risk” adj2 (re-hospitali#ation or rehospitali#ation)).ti,ab.

26. probability of repeated admission.ti,ab.

27. 11 or 12 or 13 or 14 or 15 or 16 or 17 or 18 or 19 or 20 or 21 or 22 or 23 or 24 or 25 or 26

28. 10 or 27

29. aged.mp. or Aged/

30. geriatrics.ti,ab. or Geriatrics/

31. (elder* or old*).ti,ab.

32. Aging/ or (aging or ageing).ti,ab.

33. frail elderly.ti,ab. or Frail Elderly/

34. 29 or 30 or 31 or 32 or 33

35. 28 and 34

**Embase:**

1. chronic disease scor*.ti,ab.

2. mortality risk* ind*.ti,ab.

3. ((charlson* or elixhauser* or comorbid* or co-morbid*) adj2 (index or indices)).ti,ab.

4. charlson comorbidity index/ or elixhauser comorbidity index/

5. cumulative illness rating scale*.ti,ab.

6. adjusted clinical group*.ti,ab.

7. (risk* adj2 (tool* or index or indices or score* or scale* or predict*)).ti,ab.

8. ((prognos* or predict*) adj2 (tool* or index or indices or score* or scale*)).ti,ab.

9. 1 or 2 or 3 or 4 or 5 or 6 or 7 or 8

10. valid*.ti,ab.

11. 9 and 10

12. (rxrisk* or rx risk*).ti,ab.

13. (medication* adj3 burden* ind*).ti,ab.

14. burden of illness scor*.ti,ab.

15. functional morbidity ind*.ti,ab.

16. multidimension* prognos* ind*.ti,ab.

17. silver code.ti,ab.

18. health intelligence system*.ti,ab.

19. combined predict* model*.ti,ab.

20. hospital admission risk profile*.ti,ab.

21. (predict* emergency admission* adj3 next year*).ti,ab.

22. predictive risk stratification model*.ti,ab.

23. (qadmission* or q-admission*).ti,ab.

24. (sparra or “scottish patients at risk of readmission and admission” or “scottish patients at risk of re-admission and admission”).ti,ab.

25. sussex predictor of key event*.ti,ab.

26. (“patients at risk” adj2 (re-hospitali#ation or rehospitali#ation)).ti,ab.

27. probability of repeated admission.ti,ab.

28. 11 or 12 or 13 or 14 or 15 or 16 or 17 or 18 or 19 or 20 or 21 or 22 or 23 or 24 or 25 or 26 or 27

29. aged.mp. or Aged/

30. elderly.mp.

31. old*.mp.

32. geriatric.mp. or Geriatrics/

33. frail*.mp.

34. 29 or 30 or 31 or 32 or 33

35. 28 and 34

**Cochrane library:**

1. chronic next disease next scor*:ti,ab

2. mortality next risk* next ind*:ti,ab

3. ((charlson* or elixhauser* or comorbid* or co-morbid*) near/2 (index or indices)):ti,ab

4. cumulative next illness next rating next scale*:ti,ab

5. adjusted next clinical next group*:ti,ab

6. (risk* near/2 (tool* or index or indices or score* or scale* or predict*)):ti,ab

7. ((prognos* or predict*) near/2 (tool* or index or indices or score* or scale*)):ti,ab

8. {or #6-#7}

9. valid*:ti,ab

10. #8 and #9

11. {or #1-#5}

12. (rxrisk* or rx next risk*):ti,ab

13. (medication* near/3 burden* next ind*):ti,ab

14. burden next of next illness next scor*:ti,ab

15. functional next morbidity next ind*:ti,ab

16. multidimension* next prognos* next ind*:ti,ab

17. silver next code:ti,ab

18. health next intelligence next system*:ti,ab

19. combined next predict* next model*:ti,ab

20. hospital next admission next risk next profile*:ti,ab

21. (predict* next emergency next admission* near/4 year*):ti,ab

22. predictive next risk next stratification next model*:ti,ab

23. (qadmission* or q next admission*):ti,ab

24. (sparra or “scottish patients at risk of readmission and admission” or “scottish patients at risk of re-admission and admission”):ti,ab

25. sussex next predictor next of next key next event*:ti,ab

26. (“patients at risk” near/2 (re-hospitalization or rehospitalization or re-hospitalisation or rehospitalisation)):ti,ab

27. probability of repeated admission:ti,ab

28. {or #10-#27}

29. MeSH descriptor: [Aged] in all MeSH products

30. #28 and #29

**Figure S1.** Summary of risk of bias and applicability assessment

RoB: Risk of bias.

**Table S1.** PRISMA (Preferred Reporting Items for Systematic Reviews and Meta-Analyses) checklist

| **Section and Topic** | **Item #** | **Checklist item** | **Location where item is reported** |
| --- | --- | --- | --- |
| **TITLE** | | |  |
| Title | 1 | Identify the report as a systematic review. | Page 1 |
| **ABSTRACT** | | |  |
| Abstract | 2 | See the PRISMA 2020 for Abstracts checklist. | Page 1 |
| **INTRODUCTION** | | |  |
| Rationale | 3 | Describe the rationale for the review in the context of existing knowledge. | Page 2 |
| Objectives | 4 | Provide an explicit statement of the objective(s) or question(s) the review addresses. | Pages 2 and 3 |
| **METHODS** | | |  |
| Eligibility criteria | 5 | Specify the inclusion and exclusion criteria for the review and how studies were grouped for the syntheses. | Page 3 |
| Information sources | 6 | Specify all databases, registers, websites, organisations, reference lists and other sources searched or consulted to identify studies. Specify the date when each source was last searched or consulted. | Pages 3 and 4 |
| Search strategy | 7 | Present the full search strategies for all databases, registers, and websites, including any filters and limits used. | Supplementary Box 1 |
| Selection process | 8 | Specify the methods used to decide whether a study met the inclusion criteria of the review, including how many reviewers screened each record and each report retrieved, whether they worked independently, and if applicable, details of automation tools used in the process. | Pages 3 and 4 |
| Data collection process | 9 | Specify the methods used to collect data from reports, including how many reviewers collected data from each report, whether they worked independently, any processes for obtaining or confirming data from study investigators, and if applicable, details of automation tools used in the process. | Page 4 |
| Data items | 10a | List and define all outcomes for which data were sought. Specify whether all results that were compatible with each outcome domain in each study were sought (e.g., for all measures, time points, analyses), and if not, the methods used to decide which results to collect. | Page 4 |
|  | 10b | List and define all other variables for which data were sought (e.g., participant and intervention characteristics, funding sources). Describe any assumptions made about any missing or unclear information. | Page 4 |
| Study risk of bias assessment | 11 | Specify the methods used to assess risk of bias in the included studies, including details of the tool(s) used, how many reviewers assessed each study and whether they worked independently, and if applicable, details of automation tools used in the process. | Page 4 |
| Effect measures | 12 | Specify for each outcome the effect measure(s) (e.g., risk ratio, mean difference) used in the synthesis or presentation of results. | Pages 4 and 5 |
| Synthesis methods | 13a | Describe the processes used to decide which studies were eligible for each synthesis (e.g., tabulating the study intervention characteristics and comparing against the planned groups for each synthesis (item #5)). | Pages 4 and 5 |
|  | 13b | Describe any methods required to prepare the data for presentation or synthesis, such as handling of missing summary statistics, or data conversions. | Pages 4 and 5 |
|  | 13c | Describe any methods used to tabulate or visually display results of individual studies and syntheses. | Pages 4 and 5 |
|  | 13d | Describe any methods used to synthesise results and provide a rationale for the choice(s). If meta-analysis was performed, describe the model(s), method(s) to identify the presence and extent of statistical heterogeneity, and software package(s) used. | Pages 4 and 5 |
|  | 13e | Describe any methods used to explore possible causes of heterogeneity among study results (e.g., subgroup analysis, meta-regression). | Not applicable |
|  | 13f | Describe any sensitivity analyses conducted to assess robustness of the synthesised results. | Not applicable |
| Reporting bias assessment | 14 | Describe any methods used to assess risk of bias due to missing results in a synthesis (arising from reporting biases). | Not applicable |
| Certainty assessment | 15 | Describe any methods used to assess certainty (or confidence) in the body of evidence for an outcome. | Not applicable |
| **RESULTS** | | |  |
| Study selection | 16a | Describe the results of the search and selection process, from the number of records identified in the search to the number of studies included in the review, ideally using a flow diagram. | Page 5; Figure 1 |
|  | 16b | Cite studies that might appear to meet the inclusion criteria, but which were excluded, and explain why they were excluded. | Not applicable |
| Study characteristics | 17 | Cite each included study and present its characteristics. | Pages 5 and 6 |
| Risk of bias in studies | 18 | Present assessments of risk of bias for each included study. | Page 8; Table 4 |
| Results of individual studies | 19 | For all outcomes, present, for each study: (a) summary statistics for each group (where appropriate) and (b) an effect estimate and its precision (e.g. confidence/credible interval), ideally using structured tables or plots. | Tables 1 to 3 |
| Results of syntheses | 20a | For each synthesis, briefly summarise the characteristics and risk of bias among contributing studies. | Pages 5 to 7 |
|  | 20b | Present results of all statistical syntheses conducted. If meta-analysis was done, present for each the summary estimate and its precision (e.g., confidence/credible interval) and measures of statistical heterogeneity. If comparing groups, describe the direction of the effect. | Not applicable |
|  | 20c | Present results of all investigations of possible causes of heterogeneity among study results. | Not applicable |
|  | 20d | Present results of all sensitivity analyses conducted to assess the robustness of the synthesised results. | Not applicable |
| Reporting biases | 21 | Present assessments of risk of bias due to missing results (arising from reporting biases) for each synthesis assessed. | Not applicable |
| Certainty of evidence | 22 | Present assessments of certainty (or confidence) in the body of evidence for each outcome assessed. | Not applicable |
| **DISCUSSION** | | |  |
| Discussion | 23a | Provide a general interpretation of the results in the context of other evidence. | Page 8 |
|  | 23b | Discuss any limitations of the evidence included in the review. | Page 9 |
|  | 23c | Discuss any limitations of the review processes used. | Page 9 |
|  | 23d | Discuss implications of the results for practice, policy, and future research. | Pages 9 and 10 |
| **OTHER INFORMATION** | | |  |
| Registration and protocol | 24a | Provide registration information for the review, including register name and registration number, or state that the review was not registered. | Page 3 |
|  | 24b | Indicate where the review protocol can be accessed, or state that a protocol was not prepared. | Page 3 |
|  | 24c | Describe and explain any amendments to information provided at registration or in the protocol. | Not applicable |
| Support | 25 | Describe sources of financial or non-financial support for the review, and the role of the funders or sponsors in the review. | Title page |
| Competing interests | 26 | Declare any competing interests of review authors. | Title page |
| Availability of data, code, and other materials | 27 | Report which of the following are publicly available and where they can be found; template data collection forms; data extracted from included studies; data used for all analyses; analytic code; any other materials used in the review. | Not applicable |

**Table S2.** Further details on model characteristics

| **Author, year** | **Prediction model** | **Predictor(s)** | **Discrimination** | **Calibration** | **Overall performance/**  **Reclassification/**  **Clinical usefulness** |
| --- | --- | --- | --- | --- | --- |
| Fan, 2006 | Triage Risk Screening Tool for Elderly Patients | Cognitive impairment; self-reported difficulty in walking or transferring; the use of 5 or more medications; an ED visit within the previous 30 days or a hospital admission within the previous 90 days, and “ED nurse concerns”.  “ED nurse concerns” is defined as presence of suspected abuse/neglect/self-neglect/exploitation, medication non-compliance if using fewer than 5 medications, suspected substance abuse, problems meeting instrumental activities of daily living, and other concerns.  Problems meeting instrumental activities of daily living is defined as difficulties with any of the following: obtaining prescriptions, obtaining food, transportation, cleaning the house, or personal hygiene. | Positive likelihood ratio (1-month): 1.03 (95%CI 0.11 to 1.96)  Negative likelihood ratio (1-month): 0.98 (95%CI 0.11 to 1.86)  Positive likelihood ratio (4-months): 1.81 (95%CI 0.74 to 2.10)  Negative likelihood ratio (4-months): 0.27 (95%CI 0.03 to 1.25) | Not reported | Not reported |
| Greenwald, 2022 | Risk Stratification Index 3.0 | Including all conditions documented in the ICD-10 | AUC: 0.79 (95%CI 0.79 to 0.79) | Calibration plot available with metrics and result interpretation: “The performance of this model is close to ideal for approximately 99% of the population.”  Observed-to-expected ratio: 0.98  Calibration estimates (intercept/slope): 0.00/1.00 | Not reported |
| Mayo, 2005 | Quan-Charlson Comorbidity Index (+ age; sex) | ICD-10 diagnosis of the following: myocardial infarction; congestive heart failure; peripheral vascular disease; cerebrovascular disease; dementia; chronic pulmonary disease; rheumatoid disease; peptic ulcer disease; mild liver disease; hemiplegia or paraplegia; renal disease; diabetes with complications; diabetes without chronic complications; any malignancy; moderate or severe liver disease; metastatic solid tumour; acquired immunodeficiency syndrome | Harrell’s *c* statistic (administrative data only): 0.72  Harrell’s *c* statistic (administrative & clinical chart data): 0.72 | Not reported | Not reported |
| O’Caoimh, 2023 | Clinical Frailty Scale | 9-point clinical measure of frailty | AUC: 0.68 (95%CI 0.59 to 0.77) | Not reported | Not reported |
| O’Caoimh, 2023 | Identification of Seniors At Risk | Need help before and after illness; previous hospital admission; presence of cognitive and visual impairments; polypharmacy (use of 3+ drugs) | AUC: 0.64 (95%CI 0.53 to 0.75) | Not reported | Not reported |
| O’Caoimh, 2023 | Programme of Research to Integrate Services for the Maintenance of Autonomy 7 | Age; sex; general health; social support; receiving care on a daily basis; physical limitation; activities of daily living | AUC: 0.66 (95%CI 0.57 to 0.75) | Not reported | Not reported |
| O’Caoimh, 2023 | Risk Instrument for Screening in the Community (Global score) | Perceived chance of the following: Institutionalisation; mortality; hospitalisation | AUC: 0.70 (95%CI 0.60 to 0.80) | Not reported | Not reported |
| O’Caoimh, 2023 | Risk Instrument for Screening in the Community (Overall score) | Perceived chance of the following: Institutionalisation; mortality; hospitalisation | AUC: 0.73 (95%CI 0.64 to 0.82) | Not reported | Not reported |
| Zekry, 2012 | Geriatric Index of Comorbidity | Ischemic or organic heart diseases; primary arrhythmias; heart diseases with a non-ischemic or non-organic origin; hypertension; stroke; peripheral vascular diseases; diabetes mellitus; anaemia; gastrointestinal diseases; hepatobiliary diseases; renal diseases; respiratory diseases; Parkinsonism and non-vascular neurologic diseases; musculoskeletal disorders; malignancies | Specificity: 99.7%  Positive predictive value: 50.0%  Negative predictive value: 72.2% | Not reported | Pseudo-*R*^2^: 0.06 (with no conclusion on overall performance) |

AUC: Area under the receiver operating characteristic curve; CI: Confidence interval; ED: Emergency department; ICD-10: Tenth edition of the International Classification of Diseases.
